# The effect of macroeconomic shocks on non-performing loans and credit risk in the iranian banking system using time-varying parameter vector autoregressions

**DOI:** 10.1371/journal.pone.0329587

**Published:** 2025-08-07

**Authors:** Pejman Peykani, Mostafa Sargolzaei, Camelia Oprean-Stan, Hamidreza Kamyabfar, Atefeh Reghabi

**Affiliations:** 1 Department of Industrial Engineering, Faculty of Engineering, Khatam University, Tehran, Iran; 2 Department of Finance and Banking, Faculty of Management and Accounting, Allameh Tabataba’i University, Tehran, Iran; 3 Faculty of Economic Sciences, Lucian Blaga University of Sibiu, Sibiu, Romania; University of Nigeria, NIGERIA

## Abstract

The increase in macroeconomic uncertainty leads to inefficiency in the financial and banking sectors, resulting in a rise in Non-Performing Loans (NPLs). When macroeconomic uncertainty increases, financial institutions experience higher inefficiencies, reflected in increased NPLs, and with proper management solutions, the economy can move toward sustainability. This research analyzes the effect of severe macroeconomic shocks on the NPLs of the Iranian banking system using the Time-Varying Parameter Vector Autoregressions (TVP-VAR) model and a Panel Data Model. The study utilizes data from 2007 to 2021 on key macroeconomic indicators such as economic growth rate, inflation rate, interest rate, unemployment rate, and exchange rate, along with the ratio of Non-Current Claims to Total Facilities as an index of credit risk and the ratio of loans to total assets as a risk-taking index for banks. Our innovation lies in analyzing these variables dynamically, accounting for their correlation and mutual impact. The findings indicate that a 1% increase in inflation leads to a 0.0061% increase in NPLs, while a 1% rise in the unemployment rate results in a 0.0182% increase in NPLs. Conversely, a 1% increase in GDP growth reduces NPLs by 0.0036%. Furthermore, shocks to interest rates, exchange rates, and economic growth increase credit risk, with a 1% interest rate shock raising the default rate from 7.8% to 9.2% over time.

## 1. Introduction

Banks can foster sustainable economic development through credit and financial policies or by allocating various types of loans [1–4]. Fundamentally, the primary duty of banks in any country is to support and promote sustainable economic growth [5–8]. However, studies indicate that unfavorable economic conditions are among the main causes of financial weakening and instability in banks, exacerbating economic crises and driving the economy toward unsustainability [9–10].

In Iran, the financing role is predominantly carried out by banks, as capital markets remain underdeveloped and access to alternative funding sources is limited. A significant portion of Iranian banks—over 80%—are state-owned or have major government-affiliated shareholders. This ownership structure, combined with years of economic sanctions, inflationary pressures, and currency volatility, has led to persistent challenges in managing non-performing loans (NPLs) and credit risk [11].

Managing credit risk and improving asset quality have become key concerns for both regulators and bank executives. According to the latest reports by the Central Bank of Iran (CBI), the average NPL ratio in the Iranian banking system reached approximately 12% in recent years, which is significantly higher than international standards. In some public banks, this ratio has even surpassed 20%, indicating severe stress in loan portfolios. The situation is further exacerbated by weak credit risk assessment practices, directed lending to state-owned enterprises, and the lack of robust insolvency frameworks [12–13].

In this context, understanding the dynamics of credit risk and its relationship with macroeconomic shocks becomes vital. Analyzing how variables such as inflation, interest rates, GDP growth, and exchange rate fluctuations influence NPLs, both individually and jointly, is essential for formulating effective policies and ensuring the stability of the Iranian banking sector.

Maintaining financial stability is a key objective for economic decision-makers, and the financial stability of banks is crucial as they form the core of monetary and banking activities [14–17]. One of the primary causes of financial instability in banks is the poor quality of their loan portfolios, which increases credit risk [18–19]. A significant decline in loan portfolio quality has resulted in substantial losses for banks and, in some cases, has triggered banking crises [20–22]. Credit risk, through loan losses, adversely affects profitability and capital adequacy. An increase in this risk heightens liquidity risk and, at times, leads to bank failures or even the emergence of a bank run phenomenon [23–28].

The bankruptcy of a bank can spread to other banks through the contagion effect potentially leading to a systemic banking crisis [29–31]. In such cases, most financial institutions are unable to meet their obligations, Consequently, the entire financial system struggles to transfer resources to various economic sectors, which adversely impacts economic growth [32–34]. Therefore, both supervisory authorities and banks place significant emphasis on measuring and managing credit risk, as well as maintaining the quality of loan portfolios [35–37].

Stress tests were introduced in response to the rising financial instability in many countries to better assess the vulnerability of the financial system, particularly the banking sector, under hypothetical adverse macroeconomic scenarios [38–39]. Today, these tests are an integral component of the Financial Sector Assessment Program (FSAP) of the International Monetary Fund. They evaluate the condition of banks during a financial crisis and assist banking system regulators and banks in addressing potential future crises [40–41]. Stress tests conducted by the risk management departments of financial institutions, without accounting for macroeconomic variables, and based on the analysis of customer behavior and its impact on banks’ balance sheets, are referred to as micro stress tests in economic terminology [42–43]. On the other hand, macro stress tests are tools designed to evaluate the internal vulnerabilities of the entire financial system. They form part of the FSAP and have now become an integral component of the monitoring and analysis framework used by policymakers [44–47].

Identifying the sources of a crisis and making appropriate decisions to mitigate its impact in Iran is crucial for several reasons. First, Iran’s economy is bank-oriented, meaning any disruption within the banking sector will likely propagate to other sectors and significantly influence the country’s macroeconomic stability [17].

Additionally, given the shift in the role of governments from corporate management to supervision, it is essential to stay informed about new supervisory methods, particularly in the banking sector. Second, considering that Iran’s economy has faced significant financial challenges, such as financial and commercial sanctions and economic recessions, over the past decade, adopting modern financial risk management tools, such as stress testing, is crucial for understanding how the banking system responds to macroeconomic shocks [42–48].

Considering the limited evidence on the impact of macroeconomic shocks on non-current loans and the credit risk of Iran’s banking system, investigating this issue is of great importance. It should be noted that Iran’s economy is bank-oriented, meaning that any disruption within the banking sector will spread to other sectors and severely impact the overall economy. Additionally, the performance of banks should be thoroughly investigated, and appropriate supervision should be implemented, as poor bank performance can lead to significant economic, political, and social consequences. Another important point is that Iran’s economy has been facing various financial crises for years, including sanctions and recession. Therefore, economic decision-makers must adopt modern and appropriate financial risk management tools to mitigate these risks.

This study investigates the dynamic and time-varying relationships between macroeconomic variables using the Time-Varying Parameter Vector Autoregression (TVP-VAR) model. By analyzing the instantaneous response functions of these variables, the research identifies economic shocks and assesses their impact on the credit risk of the banking system under stress conditions. MATLAB software is employed to estimate these dynamic relationships, particularly focusing on the factors influencing banks’ loan portfolios. Additionally, the relationship between macroeconomic variables and non-performing loans is analyzed through panel data regression in EVIEWS software.

The novelty of this research lies in its innovative approach to assessing the interconnected effects of economic shocks on financial variables. Unlike traditional methods, this model enables a more comprehensive analysis by accounting for the correlated impacts of macroeconomic shocks—a crucial improvement given the typically strong interdependencies among macroeconomic variables. This advanced assessment is made possible through the application of the TVP-VAR model. Moreover, this study is among the first to evaluate banking system risks in Iran during periods of significant economic turbulence, offering valuable insights for effective risk management and policy-making under stress conditions.

The rest of this article is organized as follows: In Section 2, previous research on credit risk and Non-Performing Loans (NPL) using various models is reviewed. In Section 3, the equations of the proposed model, along with an introduction to the model’s different parameters, are analyzed. Section 4 presents the results obtained from the proposed model and evaluates the findings from the stress tests. Finally, Section 5 provides the conclusion and offers suggestions for future research.

## 2. Literature review

This section provides an overview of the applied research on crisis testing and credit risk in banks using various models.

Yurdakul [49], attempted to analyze the relationship between credit risk and macroeconomic variables such as interest rate, unemployment rate, bank NPL, and exchange rate. To achieve this, data from the Turkish banking system between 1998 and 2012 were used. The results indicate that credit risk is reduced by variables such as the growth rate, while it is increased by variables such as money supply, exchange rate, unemployment rate, inflation rate, and interest rate.

Hada et al. [50] analyzed the factors affecting NPLs with the aim of strengthening banks in Romania. To investigate this, data from the Romanian banking system during the years 2009 to 2019 were used along with linear regression. The results show that the factors influencing this variable include the unemployment rate and inflation rate. Furthermore, studies indicate that the Romanian exchange rate was a significant factor in the high growth of banks’ NPLs.

Onder et al. [51] attempted to analyze the effect of credit risk on the capital adequacy variable for the Turkish banking system by using stress tests and a panel model. The variables used to achieve this goal in the article include economic growth, interest rate changes, loan growth, and non-performing loans (NPLs). The results indicate that the relationship between economic growth and interest rate changes with loans is significant. Additionally, the relationship between economic growth, exchange rate, and unemployment rate with non-performing loans (NPLs) is also meaningful. The findings suggest that one of the key reasons for the high resilience of the Turkish banking system is its adequate core capital.

Abdolshah et al. [52] investigated the impact of economic shocks on the credit losses of banks and the amount of minimum critical capital. To achieve this, the economic data of Iranian banks and the multi-stage VAR model were used. In addition, linear and non-linear models were compared in this study. First, the relationships between the variables of the model were extracted, and then the regression probabilities were analyzed and compared with those from the linear model. Furthermore, the obtained probabilities were examined using the Monte Carlo simulation, and credit losses were calculated. The results indicate that the credit loss distribution is skewed to the right, and the estimate of the minimum critical capital value obtained from the Quantile model is higher than that obtained from the linear model. These studies, however, primarily relied on panel and linear models, potentially limiting the capture of non-linear relationships or the dynamic impact of shocks.

Other researches have paid more attention to non-linear relationships, Rakotonirainy et al. [53] sought to investigate the impact of economic shocks on the credit portfolio and investment. To achieve this, the nonlinear GVAR model and stress tests were used. Additionally, non-current loans from the Madagascar banking system were used to determine critical capital. The results, after forecasting the capital, indicate that the banking system will respond appropriately to the applied economic shocks. Furthermore, the banking system in this country is highly flexible, and the necessary investments have been made in the system.

Barra & Ruggiero [54] analyzed the variables affecting NPLs and the market structure in the Italian banking system. For this purpose, banking data from Italian banks during the years 2001 to 2014 were used. The results show that the factors directly affecting a bank’s profit are the number of branches and deposit density. Similarly, Foglia [55] aimed to analyze the factors influencing banks’ NPLs. To investigate this, economic data from the Italian banking system during the years 2008 to 2020 and the Autoregressive Distributed Lag (ARDL) Cointegration model were used. The results indicate that GDP and public debt do not have a positive effect on NPLs, unlike the unemployment rate.

Naili & Lahrichi [56] sought to investigate the factors affecting banks’ NPLs. To achieve this goal, they used economic data from reliable banks in the MENA region during the years 2000–2020. Additionally, they employed the panel approach and generalized method of moments (GMM) models. The results show that factors such as GDP, capital, bank performance, and inflation have a significant impact on NPLs, unlike loan growth and banking diversity. Similarly, Okyere & Mensah [57] examined the factors affecting NPLs in banks. To achieve this, they used economic and banking data from Ghana’s banking system during the years 2007–2019, along with the ARDL bounds test. The results of this test show that factors influencing NPLs include interest rates, profitability, capital adequacy, cost-to-income ratio, inflation, and economic growth. The conclusion of this study is that bank management should focus on reducing interest rates and increasing efficiency to manage NPLs.

Dewi et al. [58] sought to evaluate the risk level of Indonesian banks using stress tests while considering macroeconomic changes. To achieve this, they used NPL data, macroeconomic data, and the Vector Autoregressive Integrated (VARI) model. The results indicate a significant relationship between macroeconomic variables and NPL variables, making it possible to predict NPLs using this model. The findings show that shocks to GDP and inflation have a critical impact on banks’ NPLs.

In an attempt to add exploring time-varying dynamics or feedback effects among variables, Alnabulsi et al. [59] sought to investigate and analyze the relationship between non-performing loans (NPLs) and banks’ profitability using the System Generalized Method of Moments (SGMM) and Panel Smooth Transition Regression (PSTR) models. To achieve this, they used economic data from 74 banks in the MENA region between 2005 and 2020. The results obtained from the linear model show an inverse relationship between NPLs and banks’ profitability. Additionally, the results from the non-linear model indicate that the relationship between NPLs and profitability remains negative, but with two distinct aspects: below 4.5%, the relationship is not significant, while above this threshold, the relationship becomes significant, however their model is not designed to quantify systemic shocks or their propagation across macroeconomic variables.

This research seeks to address these limitations by combining insights from previous studies and employing a time-varying parameter vector auto regression (TVP-VAR) model. This approach allows for the dynamic quantification of systemic shocks and their impacts on NPLs, offering a more comprehensive analysis of credit risk under macroeconomic shocks. Unlike prior studies, this research explicitly integrates these shocks into a credit risk model to evaluate the direct and correlated effects over varying time horizons, providing both practical and theoretical contributions.

Table 1, presents a detailed summary of studies conducted on credit risk and stress tests, including the location, year of implementation, proposed model, and the credit and macroeconomic variables examined in these articles.

**Table 1:** Studies on stress tests and credit risk.

Reserach	Country	Period	Model	Credit Risk Variable	Macroeconomic Variable
Pesaran et al. [60]	European Union	1979-1999	GVAR	Probability of Joint Default	GDP, CPI, Exchange Rate, Money Supply, Stock Price, Interest Rate
Virolainen [47]	Finland	1986-2003	Panel	Logit Transfer from Default Rate $Ln(\frac{1-{PD}_{it}}{{PD}_{it}})$	GDP Growth, Interest Rate, Debt-to-Asset
Hoggarth et al. [61]	England	1988-2004	VAR	Burnt Loans	GDP, Interest Rate, Exchange Rate
Castrén et al. [62]	European Union	1992-2005	GVAR	LGD (Loss Default)	GDP, Stock Market Value, inflation, Interest Rate
Foglia [63]	Italy	1991-2006	FAVAR	Default Ratio of New Loans to Total Operating Loans	GDP Growth, Labor Wages, Household Cost, Inflation
Espinoza & Prasad [64]	GCC	1995-2008	PVAR	Logit Transfer from Default Rate $Ln(\frac{1-{NPL}_{it}}{{NPL}_{it}})$	Growth Rate Without Oil, Stock Market Returns, Interest Rate
Nordal & Syed [65]	Norway	1998-2008	Panel	Logit Transfer from Weighted Default Rate $Ln(\frac{1-{DWPD}_{t}}{{DWPD}_{t}})$	GDP Growth, Exchange Rate, Household Wage
Tian & Yang [66]	China	1985-2008	VAR	Non-Performing Loans	GDP Growth, Nominal Interest Rate, CPI, Unemployment Rate
Vazquez et al. [67]	Brazil	2001-2009	Panel/ VAR	Logit Transfer from Default Rate $Ln(\frac{1-{NPL}_{it}}{{NPL}_{it}})$	GDP Growth, Bank-Level Fixed Effects
Yurdakul [49]	Turkey	1998-2012	General to Specific Modelling	Non-Performing Loans	Inflation Rate, Interest Rate, the ISE-100 Index, Foreign Exchange, Growth R ate, Unemployment Rate
Onder et al. [51]	Turkey	2007-2014	Satellite Panel	Capital Adequacy	Economic Growth, Exchange Rate, Unemployment Rate
Hada et al. [50]	Romania	2009-2019	Linear Reg.	Non-Performing Loans	Exchange, Unemployment and Inflation Rates
Barra & Ruggiero [57]	Italy	2001-2014	Linear Reg.	Non-Performing Loans	Branch Density, Deposit Density and Specialisation
Foglia [55]	Italy	2008-2020	ARDL	Non-Performing Loans	GDP Growth, Public Debt, Unemployment Rate and Domestic Credit
Naili & Lahrichi [56]	MENA	2000-2019	GMM	NPL Logit Transformation	GDP Growth, Unemployment, Bank Capitalization, Inflation, Sovereign Debt and Bank Size
Okyere & Mensah [57]	Ghana	2007-2019	ARDL	Non-Performing Loans	Inflation and Economic Growth, Lending Rate, Cost to Income Ratio, Capital Adequacy Ratio and Net Interest Margin
Alnabulsi et al. [59]	MENA	2005-2020	SGMM/ PSTR	Non-Performing Loans	GDP Growth, Inflation
**Our Research**	Iran	2007-2021	TVP-VAR	Non-Performing Loans	Interest Rate, Inflation, Gross Domestic Product, Unemployment Rate, Exchange Rate

In Table 1, both international and Iranian studies on credit risk and stress tests using various models, such as the VAR model, are presented. While numerous studies have analyzed the impact of macroeconomic shocks on Non-Performing Loans (NPLs) and the credit risk of banking systems, none of these studies have applied the Time-Varying Parameter Vector Autoregressions (TVP-VAR) model using data from Iran’s banking system. To fill this gap, this research explores, for the first time, the effect of macroeconomic shocks on NPLs in Iran’s banking system using the TVP-VAR model and economic data specific to Iran.

## 3. Methodology

In this section, the equations used in the proposed model, the model’s conditions, and an introduction to the variables are presented. Regarding the research method, this study is descriptive-analytical and investigates the effect of macroeconomic shocks (interest rate, inflation, GDP, unemployment rate, and exchange rate) on the banking system from the perspective of the credit channel using regression analysis.

In order to build a theoretical framework linking macroeconomic shocks to non-performing loans (NPLs) and credit risk, we focus on the channels through which these variables impact the repayment capacity of borrowers, thereby influencing the level of credit risk and the ratio of non-performing loans in the banking system. One of the core elements of the framework is to illustrate how economic shocks affect borrowers’ ability to service their debts.

For instance, an increase in interest rates typically raises the cost of borrowing, making it more difficult for borrowers to meet their obligations. This leads to a rise in non-performing loans as more borrowers default on their loans due to financial strain. Similarly, inflation erodes the purchasing power of borrowers’ incomes, which can reduce their ability to repay loans, especially if their income does not increase in line with rising prices. This dynamic often results in higher NPLs as borrowers struggle with higher living costs [68–69].

Another critical aspect of the framework is the relationship between economic growth (or contraction) and credit risk. A decline in GDP growth often signals an economic downturn, leading to higher unemployment rates and lower household incomes, which significantly affect borrowers’ repayment capacity. In such conditions, the likelihood of default increases as businesses reduce investment and employment, leading to widespread economic stress.

The exchange rate also plays a crucial role in the theoretical framework. A depreciation in the local currency increases the cost of imported goods and services, leading to higher overall inflation. For borrowers with loans denominated in foreign currencies, this increases the burden of debt repayment. Furthermore, exchange rate fluctuations can affect the balance sheets of banks, particularly in emerging markets, as significant exposure to foreign currency risk can destabilize the financial system. While the direct relationship between exchange rate volatility and NPLs may vary, unpredictable currency movements can contribute to economic instability, raising the likelihood of defaults and higher credit risk [70–71].

At the heart of this theoretical framework is the idea that macroeconomic shocks do not act in isolation but often have cumulative effects that amplify financial distress in the banking sector. For example, an interest rate shock may lead to an immediate increase in the cost of credit, but its effects may be exacerbated by a concurrent rise in inflation or a slowdown in economic growth. Over time, the combined impact of these shocks can significantly increase the default rate on loans and non-performing loans (NPLs).

In this study, we incorporate the assessment of macroeconomic shocks within a framework that evaluates the dynamic interactions among various variables. With this contribution, we aim to address the gap in previous research by considering these complex dynamics.

**Fig 1:**
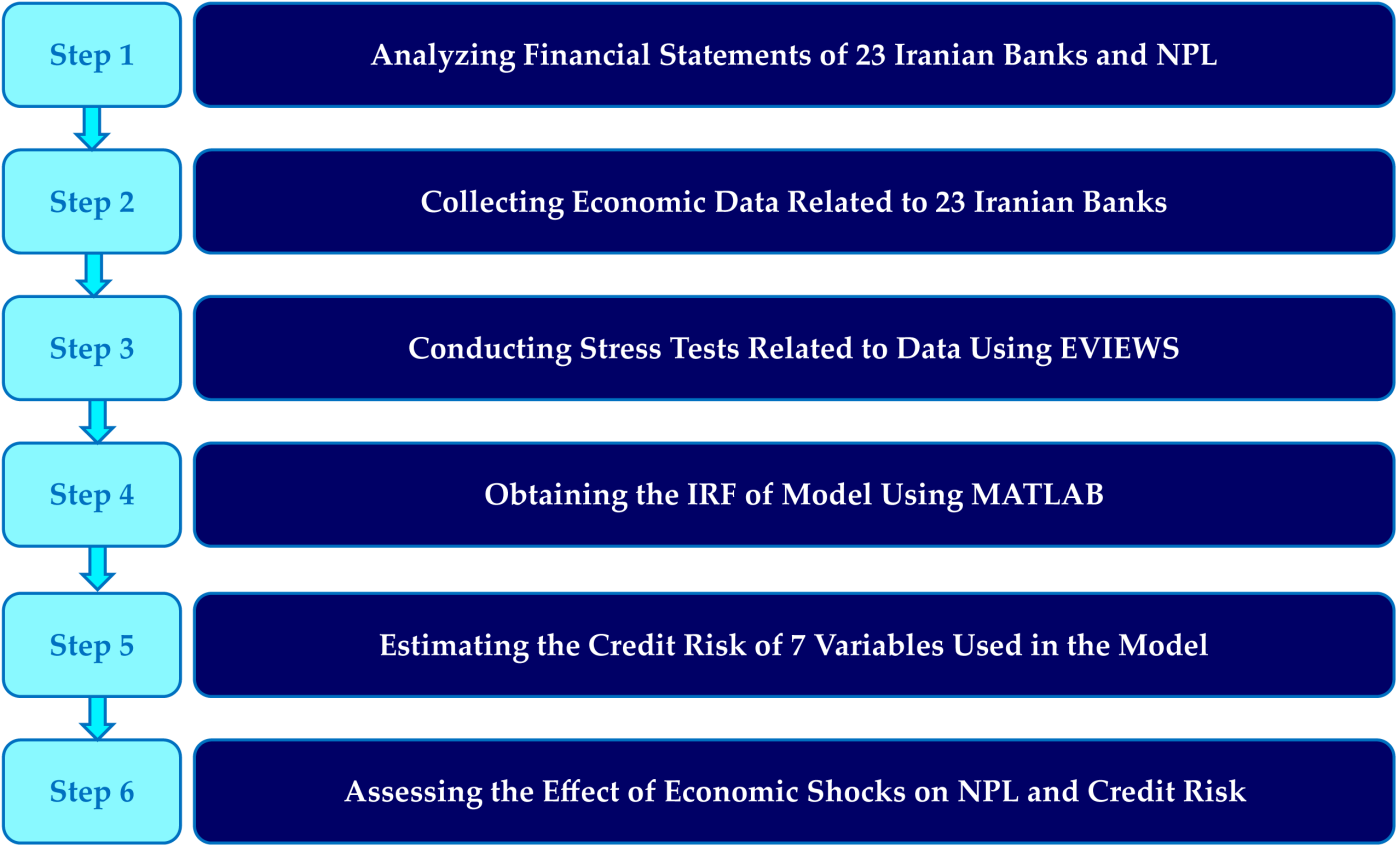
Schematic overview of the steps in the proposed TVP-VAR model.

A schematic summary of the steps of the model is provided in Fig 1.

In this research, a Time-Varying Parameter Vector Autoregression (TVP-VAR) model is used to analyze macroeconomic variables and extract shocks. VAR models are fundamental econometric tools with wide applications in econometric analysis. Among these, the TVP-VAR model, which accounts for stochastic fluctuations, is extensively used in the analysis of macroeconomic issues [72–74]. The TVP-VAR model allows for the consideration of the changing structure of the economy over time in a flexible and robust manner. However, economic variables often exhibit trends that are influenced by random fluctuations, which, when paired with time-varying coefficients but constant fluctuations, can lead to biased estimates of the variable coefficients. To address this issue, the TVP-VAR model assumes that the fluctuations are random. While random fluctuations complicate estimation due to the complexity of the likelihood function, the model can be estimated using the Markov Chain Monte Carlo (MCMC) method. According to [75], the regression model used in this research to estimate the relationship between financial and macroeconomic variables affecting the banks’ loan portfolio is as follows:


$${rGDP}_{t}=\beta_{10.t}+\sum_{n=1}^{k}\gamma_{1n.t}{rGDP}_{t-a}+\sum_{m=1}^{k}\delta_{1m.t}{HICP}_{t-m}+\sum_{d=1}^{k}c_{1d.t}{IR}_{t-d}+\sum_{b=1}^{k}\varphi_{1b.t}U_{t-b}+\sum_{e=1}^{k}\rho_{1e.t}{REERgr}_{t-e}+\varepsilon_{1t}$$ (1)

$${HICP}_{t}=\beta_{20.t}+\sum_{n=1}^{k}\gamma_{2n.t}{rGDP}_{t-a}+\sum_{m=1}^{k}\delta_{2m.t}{HICP}_{t-m}+\sum_{d=1}^{k}c_{2d.t}{IR}_{t-d}+\sum_{b=1}^{k}\varphi_{2b.t}U_{t-b}+\sum_{e=1}^{k}\rho_{2e.t}{REERgr}_{t-e}+\varepsilon_{2t}$$ (2)

$${IR}_{t}=\beta_{30.t}+\sum_{n=1}^{k}\gamma_{3n.t}{rGDP}_{t-a}+\sum_{m=1}^{k}\delta_{3m.t}{HICP}_{t-m}+\sum_{d=1}^{k}c_{3d.t}{IR}_{t-d}+\sum_{b=1}^{k}\varphi_{3b.t}U_{t-b}+\sum_{e=1}^{k}\rho_{3e.t}{REERgr}_{t-e}+\varepsilon_{3t}$$ (3)

$$U_{t}=\beta_{40.t}+\sum_{n=1}^{k}\gamma_{4n.t}{rGDP}_{t-a}+\sum_{m=1}^{k}\delta_{4m.t}{HICP}_{t-m}+\sum_{d=1}^{k}c_{4d.t}{IR}_{t-d}+\sum_{b=1}^{k}\varphi_{4b.t}U_{t-b}+\sum_{e=1}^{k}\rho_{4e.t}{REERgr}_{t-e}+\varepsilon_{4t}$$ (4)

$${REER}_{t}=\beta_{50.t}+\sum_{n=1}^{k}\gamma_{5n.t}{rGDP}_{t-a}+\sum_{m=1}^{k}\delta_{5m.t}{HICP}_{t-m}+\sum_{d=1}^{k}c_{5d.t}{IR}_{t-d}+\sum_{b=1}^{k}\varphi_{5b.t}U_{t-b}+\sum_{e=1}^{k}\rho_{5e.t}{REERgr}_{t-e}+\varepsilon_{5t}$$ (5)

In Equations (1) to (5), the variables represent the following,

- $\beta_{i0.t}$: Intercept term representing the baseline effect before considering explanatory variables,
- $\gamma_{in.t}:\;$ Coefficients capturing the impact of lagged values of real GDP (rGDP) on the dependent variables,
- rGDP: Percentage change in real GDP compared to the previous year,
- $\delta_{im.t}:\;$ Coefficients representing the influence of lagged values of the inflation rate (HICP),
- HICP: Percentage change in the consumer price index,
- $c_{4d.t}:\;$ Coefficients measuring the effect of interest rate (IR) on each dependent variable,
- IR: Interest rate,
- $\varphi_{ib.t}:\;$ Coefficients capturing the effect of unemployment rate (U) over different time periods,
- U: Unemployment rate,
- $\rho_{5e.t}:\;$ Coefficients estimating the effect of exchange rate growth (REER) over various lagged periods,
- REER: Exchange rate, defined as the value of the US dollar against the Rial,
- $\epsilon_{5t}:\;$ Error term accounting for unobserved shocks in each equation.

In the regression presented in this research, widespread systemic shocks are considered, which cause the economic growth rate, inflation rate, interest rate, unemployment rate, and exchange rate to interact with each other and be influenced by both their own shocks and those of other macroeconomic variables. Additionally, it is assumed that all parameters follow a first-order random process, which allows for both temporary and permanent changes in the parameters. Next, as done by [75] the credit risk of the banking system is a function of five common macroeconomic variables in explaining the default rate: GDP growth, inflation rate changes, exchange rate changes, interest rate changes, and unemployment rate changes, as well as changes in the ratio of banks’ loans to assets. The loan-to-asset ratio is considered an indicator of banks’ risk-taking behavior, and as this ratio increases, credit risk in banks also increases.


$${NPL}_{i.t}=\alpha_{0}+\alpha_{1}{RGDP}_{t}+\alpha_{2}{DHICP}_{t}+\alpha_{3}{DUnem}_{t}+\alpha_{4}{DLoantoasset}_{i.t}+\alpha_{5}{IR}_{t}+\alpha_{6}{REER}_{t}+\vartheta_{i.t}$$ (6)

In Equation (6), the variables represent, respectively:

- $\alpha_{0}$: Constant term in the regression model,
- $\alpha_{1}-\alpha_{6}$: Regression coefficients capturing the influence of each independent variable (macroeconomic shocks) on the NPL ratio,
- ${NPL}_{i.t}$ the percentage ratio of non-performing loans to the total facilities of bank i at time t,
- ${RGDP}_{t}$: real GDP growth at time t,
- ${DHICP}_{t}$: the change in inflation at time t,
- ${DUnem}_{t}$: the change in unemployment rate at time t,
- ${DLoantoasset}_{i.t}$: the change in the ratio of total loans to total assets of bank i at time t,
- ${IR}_{t}\;:$ the change in interest rate at time t,
- ${REER}_{t}\;:$ the growth of exchange rate at time t,
- $\vartheta_{i.t}\;:$ Error term accounting for unobserved factors affecting the NPL ratio.

The estimation of Equation (6) using the variable data would be meaningless, as the defined model cannot adequately explain the changes in the dependent variable. To address this issue, the first-order differences of the inflation rate, unemployment rate, loan-to-total-assets ratio, and interest rate, along with the economic growth rate and currency growth rate, are used to ensure the regression model’s validity. Additionally, in the panel data method, the length of the time series is significant. To avoid the formation of a spurious regression, the stationarity of the variables is typically verified. However, since the number of years considered in this research (15 years) is below the threshold required for stationarity checks, the stationarity of the variables will not be tested.

At the final step of the methodology in this study, macroeconomic shocks are explicitly incorporated into the credit risk model. These shock values are integrated into the banking system’s credit risk model, specifically within the equation estimating the non-performing loan (NPL) ratio. As described earlier, the shock values are extracted and quantified based on the time-varying parameter vector autoregression (TVP-VAR) model.

This approach enables the analysis of how each shock individually affects the banking system’s credit risk, while also accounting for its potential influence on other variables over one, six, and twelve periods following each shock.

## 4. Case study

In this section, the proposed model is evaluated using financial and economic data from Iran’s banking system. A real dataset specific to Iran’s banking sector has been utilized for this purpose.

The statistical population examined in this research comprises 23 banks listed on the Iran Stock Exchange. Data on non-current claims (Overdue, Outstanding, and Bad Debts) as a percentage of total facilities, representing the banks’ credit risk index, and the ratio of loans to total assets, indicating risk-taking behavior, were collected annually from 2007 to 2021.

The choice of the 2007–2021 period is deliberate and empirically justified, as it encompasses major economic events affecting Iran’s banking sector, including the global financial crisis, multiple rounds of economic sanctions, and the COVID-19 pandemic. These events introduced considerable volatility in macroeconomic indicators such as inflation, interest rates, and the exchange rate—making the period ideal for analyzing dynamic credit risk behavior. Moreover, this time frame aligns with improved data availability and regulatory transparency in the Iranian banking system. The length and turbulence of the period also suit the TVP-VAR methodology, which requires a sufficient time span to capture structural changes in macro-financial linkages.

These data extracted from the financial statements of governmental and non-governmental banks available on the Codal website (www.codal.ir), the platform for publishing data on firms listed in the Iran Stock Exchange Organization. Additionally, macroeconomic data, except for the unemployment rate (sourced from the Statistical Center of Iran’s website), were obtained from the Central Bank of Iran’s time series database (www.cbi.ir).

According to the reports of the Central Bank of Iran, the volume of non-current claims of Iran’s banking system has increased 45 times between 2007 and 2021. An analysis of the ratio of non-current facilities to total facilities over the past decade shows an upward trend in this index until 2011, followed by a consistent downward trajectory thereafter.

To validate the presented regression, a stationarity test must first be conducted. Table 2 presents the results of the augmented Dickey-Fuller test, which evaluates the stationarity of the macroeconomic variables used in the Vector Autoregression model. The results indicate that GDP growth, the inflation rate, and the interest rate are stationary at their levels.

**Table 2:** Augmented Dickey–Fuller Test.

Variable		rGDP	HICP	IR	REER	U
**At Level**	**Intercept**	−3.65(0.01)	−2.76(0.07)	−2.47(0.13)	0.15(0.96)	−2.31(0.17)
	**Intercept and Trend**	−3.72(0.03)	−2.85(0.19)	−3.53(0.05)	−1.45(0.81)	−1.93(0.59)
**At First Difference**	**Intercept**	–	–	–	−2.89(0.05)	−3.31(0.07)

In Table 2, the first row of numbers represents the t-statistic values of the variables, while the second row contains their corresponding probabilities (Prob). Since some of the variables in the model are non-stationary, there is a risk of spurious regression. One approach to address this is to stabilize the non-stationary variables by differencing them, after which the regression can be estimated using the differenced variables. However, this approach results in the loss of long-term information embedded in the variables. An alternative method is to establish the presence of a long-term equilibrium relationship among the variables. If such a relationship exists, the risk of spurious regression is eliminated, and it becomes unnecessary to stabilize the variables. In this study, the Johansen Cointegration test was employed to assess whether a long-term equilibrium relationship exists between the model variables.

According to the findings of Table 3, the presence of a long-term relationship between the variables is confirmed based on both statistical measures, eliminating the need to stabilize the variables.

**Table 3:** Johansen cointegration test.

Null Hypothesis	Eigenvalues	Trace Statistic	5% Critical Value	Prob
**None**	0.9969	216.64	69.81	0.00
**At Most 1**	0.9471	112.51	47.85	0.00
**At Most 2**	0.8706	59.57	29.79	0.00
**At Most 3**	0.7173	22.75	15.49	0.00
**At Most 4**	0.0008	0.015	3.84	0.00
**Null Hypothesis**	**Eigenvalues**	**Max Statistic**	**5% Critical Value**	**Prob**
**None**	0.9969	104.132	33.87	0.00
**At Most 1**	0.9471	52.9387	27.58	0.00
**At Most 2**	0.8706	36.8196	21.13	0.00
**At Most 3**	0.7173	22.7423	14.26	0.00
**At Most 4**	0.0008	0.0152	3.84	0.00

## 4. Experimental discussion

**Fig 2:**
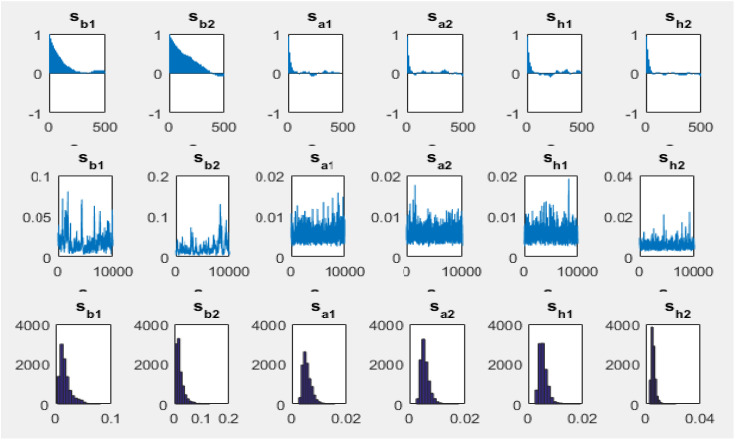
Autocorrelation, sampling path and posterior density of the model.

The figures of the first row in Fig 2 illustrate the autocorrelation of the variance of the error term in Equations (1) to (5). The second row of Fig 2 displays the sampling paths of selected parameters (e.g., the autocorrelation example), based on 10,000 samples. Each of these samples corresponds to the posterior density shown in the third row of Fig 2.

A high degree of autocorrelation at the initial lags is observed in some parameters, particularly s_b1 and s_b2, indicating persistent dependency structures in these variables. However, the decreasing pattern suggests that the autocorrelation effect diminishes over time, which is crucial for ensuring the stability of the model.

The second row of Fig 2 represents the sampling paths of selected parameters across 10,000 samples. These paths indicate the fluctuations and convergence behavior of the estimated parameters. The relatively stable movement of parameters such as s_a1, s_a2, s_h1, and s_h2 suggests that the model has achieved a reasonable level of convergence. However, in some parameters (s_b1, s_b2), a higher degree of variability is observed, indicating potential sensitivity to input variations or requiring a longer burn-in period in the Markov Chain Monte Carlo (MCMC) estimation.

The third row of Fig 2 shows the posterior density distributions of the sampled parameters. These densities indicate the range and distributional characteristics of each estimated parameter. The posterior densities of s_b1 and s_b2 appear right-skewed, suggesting that these parameters have higher estimated values concentrated around the lower end of the distribution. Meanwhile, the density distributions of s_a1, s_a2, s_h1, and s_h2 are more symmetric and concentrated within a narrower range, implying a more robust and stable estimation.

Table 4 presents the mean and standard deviation of each posterior density. As observed in Fig 2, after 10,000 samples, the sampling paths stabilize, and the sample autocorrelation decreases steadily, indicating that the sampling method employed in this study effectively generates samples with low autocorrelation.

**Table 4:** Estimation results.

Parameter	Mean	Stdev	95%U	95%L	Geweke	Inef.
**sb1**	0.0174	0.0134	0.0052	0.0564	0.414	141.94
**sb2**	0.0160	0.0088	0.0048	0.0374	0.492	152.84
**sa1**	0.0055	0.0016	0.0034	0.0096	0.100	23.03
**sa2**	0.0056	0.0018	0.0033	0.0101	0.966	48.04
**sh1**	0.0056	0.0017	0.0033	0.0103	0.204	34.47
**sh2**	0.0056	0.0016	0.0034	0.0094	0.860	35.23

The figures in the third row depict the posterior densities derived from the prior densities and sample data for two selected parameters. Specifically, the prior density of each parameter has been updated using 10,000 random samples and the sample data, resulting in a new density function—referred to as the posterior density—visible in the third row.

In Table 4, in addition to the mean and standard deviation, the posterior densities of the Geweke and Inefficiency statistics are presented. These are used to assess convergence and inefficiency factors, respectively. According to the estimation results, the null hypothesis (indicating convergence to the prior distribution) is not rejected at the 5% significance level based on the Geweke statistic.

Furthermore, the inefficiency factors (Inef.) are relatively low for most parameters and state variables, except for sb1 and sb2 with 141.94 and 152.84, respectively. However, given the number of samples (10,000 iterations), the sampling for these two parameters is also considered efficient.

**Fig 3:**
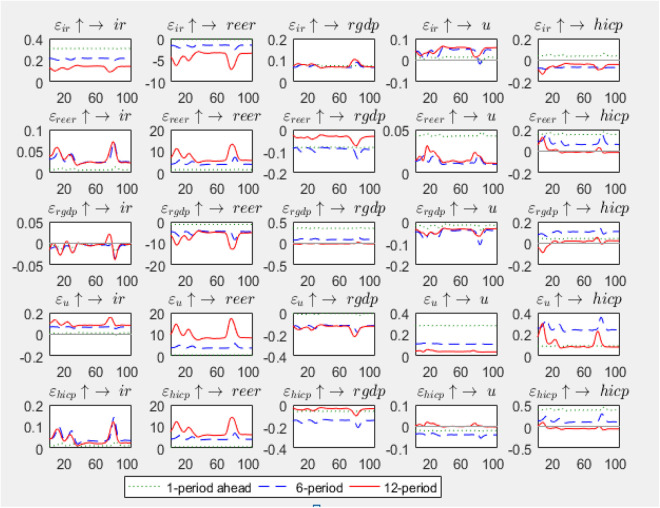
Impulse Response Function (IRF) of model variables.

The Impulse Response Function (IRF) illustrates the dynamic behavior of variables over time in response to an impulse. Fig 3 depicts the dynamic responses of the model variables to a one-standard-deviation impulse in the interest rate, exchange rate, economic growth rate, unemployment rate, and inflation rate over one, six, and twelve periods. Additionally, the time-varying coefficients of each variable’s impact on itself and on other variables are presented in this figure.

For instance, in the first row of Fig 3, the coefficient of the interest rate on itself is both positive and decreasing over three periods. This pattern occurs because, following a shock to the interest rate, the interest rate settles at progressively lower levels in subsequent periods. Similarly, the coefficient of the interest rate on the exchange rate is negative and decreasing. This reflects the negative impact of an interest rate impulse on the exchange rate. From the perspective of domestic investors, an increase in interest rates makes bank deposits more attractive, reducing the demand for foreign currency and thereby decreasing the exchange rate.

The variable coefficient of the interest rate on GDP over three periods shows minimal changes and remains positive. The impact of an interest rate impulse on GDP is also positive. This can be attributed to the fact that interest rates (on deposits and facilities) in the examined context have historically been set administratively and kept below the inflation rate, resulting in a negative real interest rate. This negative real interest rate has encouraged increased investment and economic growth, even in the presence of a positive shock to the interest rate.

The variable coefficient of the interest rate on the unemployment rate is upward and positive, indicating that an increase in the interest rate leads to a rise in unemployment. Similarly, the effect of an interest rate impulse on the unemployment rate is also positive.

The variable coefficient of the exchange rate on economic growth is positive, while the effect of an exchange rate impulse on economic growth is negative. This can be explained by the unique structure of Iran’s economy, where the supply of foreign currency is monopolized by the government, and exchange rates are not determined by market dynamics of supply and demand. As a result, the exchange rate does not reflect the actual economic strength of the country but instead imposes a set of artificial prices on the economy. Additionally, since Iran’s production and industrial sectors heavily depend on foreign sources for raw materials, equipment, and facilities, an increase in the exchange rate (devaluation of the national currency) leads to reduced economic growth. This devaluation does not significantly enhance the competitiveness of domestically produced goods, nor does it boost exports and economic growth.

The variable coefficient of economic growth on the unemployment rate is downward and negative, indicating that higher economic growth leads to a reduction in unemployment. Similarly, the effect of an economic growth impulse on the unemployment rate is also negative.

The variable coefficient of the inflation rate on the economic growth rate initially decreases and is negative, then increases but remains negative. This suggests that as the inflation rate rises, the real return on savings and investments diminishes, adversely affecting economic growth.

The results of the F-Limer test are shown in Table 5, where the null hypothesis represents the Pooled Model and the alternative hypothesis represents the Fixed Effect Model, are as follows:

**Table 5:** F-limer test.

Test	F-Statistic	DF	Probability
**Cross-Section F**	10.688	22.154	0.0000
**Cross-section Chi-square**	169.642	22.000	0.0000

The results indicate that the null hypothesis is rejected at a 99% confidence level, confirming that the Fixed Effect Model (FEM) is preferred over the Pooled Model. This suggests that banks exhibit significant heterogeneity in terms of the ratio of non-current claims. Consequently, using panel data techniques allows the model to control for unobserved heterogeneity across banks, leading to more reliable parameter estimates.

Additionally, rejecting the Pooled Model implies that assuming a common intercept across all banks would be inappropriate. Instead, the Fixed Effect Model accounts for individual bank-specific effects, which are likely influenced by factors such as institutional policies, risk management strategies, and financial structures.

These findings validate the use of the panel data estimation approach in Equation (6), ensuring that variations in non-performing loans (NPLs) and credit risk among banks are properly accounted for in the model.

After confirming the panel data method, the Hausman test is performed, as shown in Table 6, to determine the suitable estimation type—fixed effects or random effects. The null hypothesis states that the difference between the estimators is not systematic, implying that REM is preferable. Conversely, rejecting the null hypothesis suggests that FEM should be used instead.

**Table 6:** Hausman test.

Test	Chi-Sq. Statistics	Chi-Sq. DF	Probability
**Cross-Section Random**	16.197	6.000	0.0127

Given that the p-value (0.0127) is statistically significant at the 5% level, the null hypothesis is rejected. This implies that the Fixed Effects Model (FEM) is more appropriate than the Random Effects Model (REM) for this dataset.

Consequently, the estimation results of the credit risk model, using the panel data method with fixed effects, are presented in Table 7.

**Table 7:** Results of credit risk estimation.

Variable	Coefficient	Standard Deviation	T-Statistic	Prob
**C**	0.0904	0.0168	5.3676	0.0000
**DHICP**	0.0061	0.0020	3.0367	0.0028
**REER**	−0.0003	0.0002	−1.3623	0.1751
**RGDP**	−0.0036	0.0017	−2.1442	0.0336
**DUNEM**	0.0182	0.0086	2.1017	0.0372
**DLOANTOASSET**	−0.1388	0.0523	−2.6516	0.0088
**DIR**	−0.0058	0.0033	−1.7204	0.0874
**R-Squared**	0.62			
**Prob(F-Statistic)**	0.0000			

The credit risk estimation results reveal that changes in the inflation rate exhibit a positive and significant relationship at the 95% confidence level with the ratio of non-current claims to total facilities. This finding underscores the adverse impact of worsening macroeconomic conditions on the increase of overdue claims. Specifically, the reduction in real household income due to rising inflation outweighs the effect of the decrease in the real value of facilities, thereby increasing the credit risk of banks. The coefficient indicates that a one-unit increase in changes to the inflation rate will result in a 0.0061-unit rise in the ratio of non-current claims to total facilities.

The growth of the exchange rate demonstrates a negative relationship with the non-performing loan (NPL) ratio, suggesting that an increase in the growth rate of the currency enhances Iran’s international competitiveness. This, in turn, improves the quality of loans in the banking system. However, this relationship is not statistically significant.

The estimation results indicate that GDP growth has a negative and significant relationship at the 95% confidence level with the ratio of non-current claims. The negative coefficient suggests that an improvement in macroeconomic conditions and an increase in economic growth led to a reduction in overdue claims within the banking system. Specifically, the coefficient implies that a 1-unit increase in economic growth results in a 0.0036-unit decrease in the ratio of non-current claims to total payment facilities.

Changes in the unemployment rate have a positive and significant relationship with the NPL ratio at the 95% confidence level. The coefficient of this variable indicates that a 1-unit increase in the unemployment rate leads to a 0.0182-unit increase in the ratio of non-current claims to total facilities. Contrary to expectations, changes in the ratio of loans to total assets of banks have a negative and significant relationship at the 99% confidence level with the NPL ratio. Theoretically, the loan-to-asset ratio is considered an indicator of a bank’s risk-taking, and an increase in this ratio typically leads to a higher NPL ratio. The negative relationship observed here can be explained by the negative real interest rate in the banking sector. During the research period, the average real interest rate was consistently negative, leading to a lower willingness of banks to extend loans despite the high cost of capital. On the other hand, factors such as unfavorable macroeconomic conditions (inflation, recession, unemployment, etc.) and legal gaps in Iran’s economy have contributed to the increasing ratio of non-current claims, particularly in non-governmental banks.

Finally, changes in the interest rate have a negative and significant relationship with the ratio of non-current claims to total facilities at the 90% confidence level. The coefficient of this variable indicates that a 1-unit increase in interest rate changes leads to a 0.0058-unit decrease in the ratio of non-current claims to total facilities. This negative relationship can be explained by the fact that, as part of an anti-recession policy, the central bank reduced interest rates despite high inflation. This reduction led to a decrease in the real interest rate in the formal banking market, creating a gap with the real interest rate in the informal market.

Next, by using the shocks extracted for macroeconomic variables based on the TVP-VAR model and incorporating the shock values of these variables into the credit risk equation of the banking system, as estimated in Table 7, the credit risk is estimated under critical conditions.

**Fig 4:**
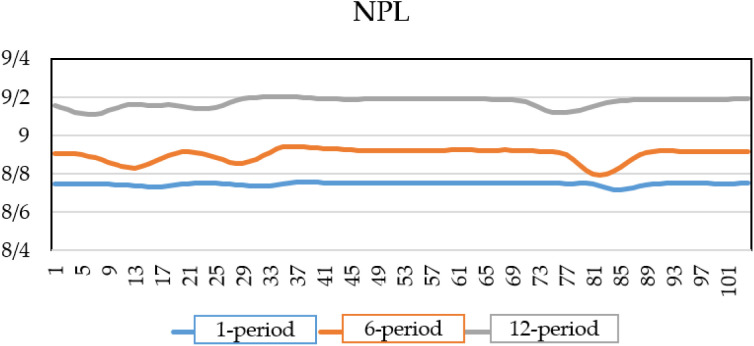
NPL of banks under interest rate shock.

As shown in Fig 4, the credit risk of the banking system increases over time when there is a shock to the interest rate. This shock, propagates through the financial system, affecting borrowing costs, investment, and overall economic stability, leads to a rise in the default rate. Initially, the default rate one period after the shock is 7.8%, Over time, as the effects of the higher interest rate spread across the banking sector, the NPL ratio increases to 8.9% after six periods and further rises to 9.2% after twelve periods.

**Fig 5:**
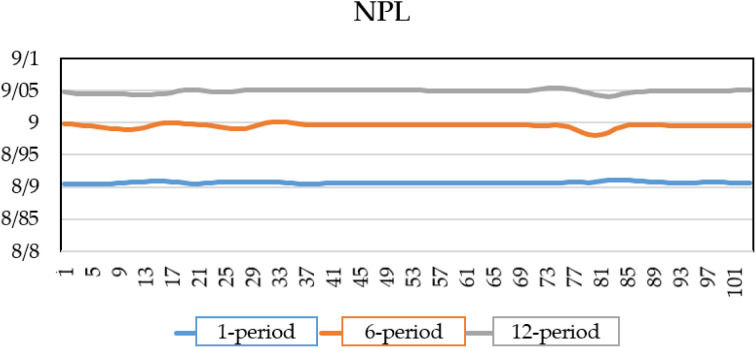
NPL of banks under exchange rate shock.

By introducing a shock to the exchange rate and considering its impact on this variable and other macroeconomic factor over three periods, the extracted shock values are incorporated into the NPL equation to estimate the credit risk of the banking system under crisis conditions for one, six, and twelve periods, as shown in Fig 5. As observed, a shock to the exchange rate leads to an increase in the banking system’s credit risk over time. Specifically, the default rate reaches 8.9% in the first period, 9% after six periods, and 9.05% twelve periods after the shock. A weaker domestic currency raises import costs, contributing to higher inflation, which in turn erodes households’ real incomes and their ability to repay loans. In addition, Currency fluctuations create economic uncertainty, discouraging investment and reducing corporate revenues, which further increases credit risk.

**Fig 6:**
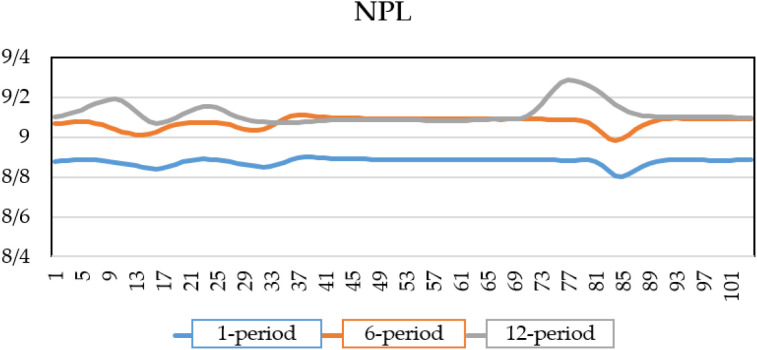
NPL of banks under GDP shock.

By introducing a shock to the economic growth rate and incorporating the shock values of this variable, along with the other macroeconomic variables affected by this shock, into the NPL ratio equation, Fig 6 illustrates that the banking system’s credit risk will reach 8.9% in the initial period. Furthermore, as the shock impacts the system over the following six (with 8.95%) and twelve periods, the credit risk will experience greater fluctuations, ultimately rising to a range of 9.1%.

**Fig 7:**
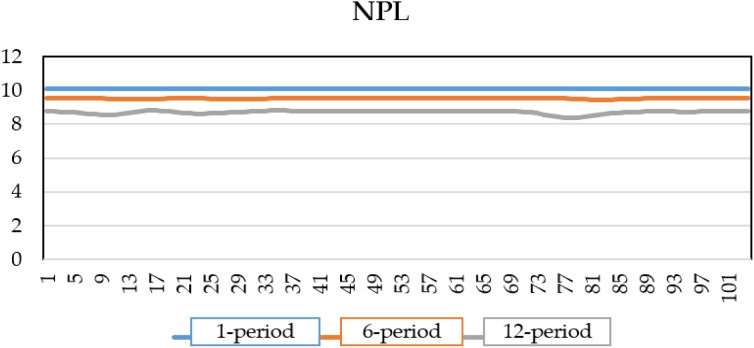
NPL of banks under unemployment rate shock.

As shown in Fig 7, by introducing a shock to the unemployment rate and incorporating the extracted shock values of the macroeconomic variables into the NPL equation, the estimated credit risk of the banking system under crisis conditions for the next one, six, and twelve periods indicates that the default rate will be at 10% just one period after the shock. As time progresses and the shock’s influence wanes, the default rate decreases, reaching 9.5% after six periods and 8.5% after twelve periods. Unlike interest rate and exchange rate shocks, which generally increase credit risk over time, an unemployment shock exhibits a different pattern, Immediate sharp increase due to borrowers’ reduced ability to meet debt obligations following job losses and Gradual decline over six and twelve periods, implying that some borrowers adjust, possibly through new employment opportunities or government interventions.

**Fig 8:**
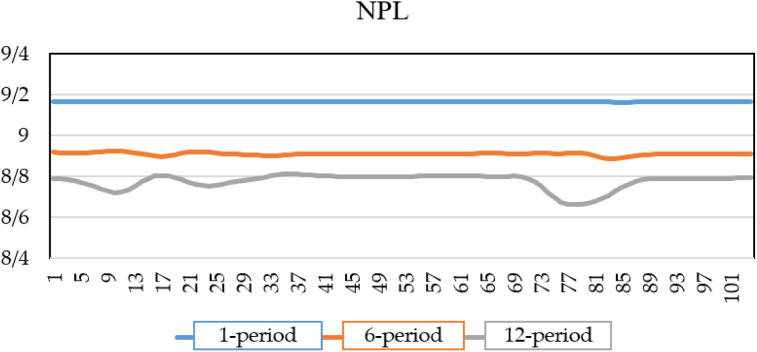
NPL of Banks under inflation rate shock.

By introducing a shock to the inflation rate and incorporating the values affected by this shock along with other macroeconomic variables for three periods into the NPL estimation equation, the credit risk of the banking system under critical conditions has been calculated for the next one, six, and twelve periods. Fig 8 shows that one period after the monetary shock, the default rate will reach 9.2%. Over time, the effect of this shock decreases, and the default rate gradually declines. In the following six periods, the NPL ratio will be 8.9%, and after twelve periods, an average of 8.8% is predicted. This could be because of customers updating with new condition.

## 5. Conclusions

In this research we assess impact of the macroeconomic shocks on banks’ performance. One of the most important methods for analyzing the performance of a financial institution is assessing its vulnerability to significant economic shocks, recognizing that the behavior of the financial system differs in normal and critical conditions. Under normal conditions, the behavior of financial institutions is relatively easier to predict, as it tends to remain stable in the short and medium term, allowing future performance to be forecasted based on past behavior. However, in critical situations, financial behaviors become unpredictable, and past performance provides limited insight into future outcomes.

From a methodological perspective, this research employs a novel approach to assess the impact of various economic shocks on bank, together with assessing macroeconomics shocks on each other which is a common phenomenon during economic crisis. This is done using a time-varying vector parameter autoregression (TVP-VAR) model, and panel data analysis.

Contrary to the findings of [49], the results of the credit risk estimation for Iranian banks indicate that most macroeconomic variables and financial indicators have a significant relationship with the banking system’s default rate. Specifically, GDP growth shows a negative and significant relationship (at the 95% level) with the ratio of non-current claims, meaning that as the economy improves, the credit risk of the banking system is expected to decrease, and vice versa. Additionally, the credit risk model reveals a positive and significant relationship (at the 95% level) between changes in the inflation rate and the NPL ratio. This suggests that the decline in household income due to rising inflation outweighs the effect of reduced real value of facilities, thereby increasing the credit risk of banks. The exchange rate growth shows a negative relationship with the NPL ratio, but this relationship is not statistically significant, potentially due to the exogenous nature of the exchange rate. Another significant finding is the ratio of loans to total assets, which has a negative and significant relationship with the default rate at the 90% level. The results also show that shocks to the interest rate, exchange rate, and economic growth rate have led to an increase in the credit risk of Iran’s banking system, highlighting the significant impact of these macroeconomic shocks on the default rate.

This study and its proposed methodology also have significant practical implications. By demonstrating the impact of economic variables on key banking parameters, the research offers economic decision-makers valuable insights into bank performance during macroeconomic shocks. Additionally, the study provides specific guidance for policymakers.

Based on the empirical findings of this study, several policy recommendations can be made to improve the resilience of the Iranian banking system against macroeconomic shocks and Provide a comprehensive package of policy recommendations supported by other key scholarly works [76–79]. First, the significant negative relationship between GDP growth and NPLs suggests that economic expansion reduces credit risk. Thus, policies that support sustainable economic growth, such as investment incentives, employment creation, and productivity-enhancing reforms, can indirectly stabilize the banking sector by improving borrowers’ repayment capacity. Second, the positive and significant impact of inflation on the NPL ratio highlights the need for strict inflation control policies. In parallel, banks should incorporate inflation-adjusted credit scoring models to assess borrower risk more accurately during volatile price periods.

Third, although interest rate shocks increase credit risk, abrupt interest rate adjustments could destabilize the financial system. Hence, a gradual and transparent interest rate policy is recommended to allow households and businesses to adapt, particularly in an economy with a history of high inflation and currency fluctuations [80].

Fourth, the loan-to-asset ratio’s negative relationship with NPLs underlines the importance of prudential credit allocation. Regulatory authorities should monitor excessive credit expansion and encourage banks to align their lending strategies with risk-based capital requirements.

It also appears that the further development of debt securities market financing is essential for reducing the financial burden often imposed on banks. One of the prerequisites for this is the advancement of understanding and comprehension of the process of better predicting corporate default [81].

These recommendations are rooted in the empirical findings and tailored to the Iranian context, offering policymakers practical tools to manage risk, reinforce financial stability, and enhance the predictive capacity of early warning systems within the banking sector.

While prior studies have examined the impact of macroeconomic and financial development indicators on non-performing loans (NPLs), this study contributes to the literature by applying a TVP-VAR model integrated with panel data analysis specifically focused on the Iranian banking system. Unlike broader regional or multi-country analyses [82], this research captures the dynamic, time-varying effects of macroeconomic shocks on credit risk within an emerging and shock-prone economy. Furthermore, the study moves beyond static estimation and employs stress-testing based on impulse response functions, allowing for forward-looking policy insights into how banks are affected over short, medium, and long-term horizons. This methodological advancement provides a more flexible and accurate tool for risk assessment under conditions of economic instability—offering practical value for both researchers and policymakers in similar developing country contexts [83].

Our scope extends only until 2021, although we believe that the findings remain applicable to the present conditions. This is due to the fact that Iran’s current economic environment closely resembles the period covered in our research, characterized by the intensification of economic sanctions and heightened political tensions.

One of the key limitations of this research is the focus on random shocks in the presented model, while other types of economic shocks could also be explored to better understand their impact on Non-Performing Loans (NPLs) and the credit risk of the banking system. Additionally, the study relies on a specific set of macroeconomic variables, and future research could expand by incorporating other variables, such as demand, disposable income, and national income, to further investigate this topic. For future research, the Data Envelopment Analysis (DEA) approach could be employed to examine the impact of macroeconomic variables on credit risk in developing countries. Moreover, the DEA approach could be used to analyze the relationship between macroeconomic variables and non-performing loans.
